# Imaging Assessment of the Postoperative Spine: An Updated Pictorial Review of Selected Complications

**DOI:** 10.1155/2021/9940001

**Published:** 2021-05-18

**Authors:** Roberto Corona-Cedillo, Melanie-Tessa Saavedra-Navarrete, Juan-Jose Espinoza-Garcia, Alexela-Nerey Mendoza-Aguilar, Sergey K. Ternovoy, Ernesto Roldan-Valadez

**Affiliations:** ^1^Neuroimaging Department, National Institute of Neurology and Neurosurgery, Mexico City, Mexico; ^2^Facultad de Ciencias de la Salud, Universidad Anahuac Mexico, Mexico City, Mexico; ^3^Facultad de Medicina, Universidad Autónoma de Coahuila, Torreo City, Mexico; ^4^Directorate of Research, Hospital General de Mexico “Dr. Eduardo Liceaga”, Mexico City, Mexico; ^5^Department of Radiology, I.M. Sechenov First Moscow State Medical University (Sechenov University), 119992 Moscow, Russia; ^6^A.L. Myasnikov Research Institute of Clinical Cardiology of National Medical Research Center of Cardiology of the Ministry of Health of Russia, 127005 Moscow, Russia

## Abstract

Imaging of the postoperative spine requires the identification of several critical points by the radiologist to be written in the medical report: condition of the underlying cortical and cancellous bone, intervertebral disc, and musculoskeletal tissues; location and integrity of surgical implants; evaluation of the success of decompression procedures; delineation of fusion status; and identification of complications. This article presents a pictorial narrative review of the most common findings observed in *noninstrumented* and *instrumented* postoperative spines. Complications in the *noninstrumented* spine were grouped in *early* (hematomas, pseudomeningocele, and postoperative spine infection) and *late* findings (arachnoiditis, radiculitis, recurrent disc herniation, spinal stenosis, and textiloma). Complications in the *instrumented* spine were also sorted in *early* (hardware fractures) and *late* findings (adjacent segment disease, hardware loosening, and implant migration). This review also includes a short description of the most used diagnostic techniques in postoperative spine imaging: plain radiography, ultrasound (US), computed tomography (CT), magnetic resonance (MR), and nuclear medicine. Imaging of the postoperative spine remained a challenging task in the early identification of complications and abnormal healing process. It is crucial to consider the advantages and disadvantages of the imaging modalities to choose those that provide more accurate spinal status information during the follow-up. Our review is directed to all health professionals dealing with the assessment and care of the postoperative spine.

## 1. Introduction

Imaging assessment of the postoperative spine requires knowledge of several key points in the medical history of the patient: anatomy and age of the patient, initial spinal pathologic condition, surgical procedure performed, clinical presentation, time interval from the surgery to the imaging study, and duration and nature of the postsurgical syndrome. Other key points that should be recognized during the imaging assessment include the condition of the underlying cortical and cancellous bone, intervertebral disc, and musculoskeletal tissues; location and integrity of surgical implants; evaluation of the success of decompression procedures; delineation of fusion status; and identification of complications [1, 2].

This article is aimed at presenting a pictorial review of some of the most common complications observed in the postoperative spine. This review presents a short description of the most used diagnostic techniques: plain radiography, ultrasound (US), computed tomography (CT), magnetic resonance (MR), and nuclear medicine. A brief description of postoperative findings is grouped in the noninstrumented spine (early and late) and instrumented spine (early and late). Our review is directed to all health professionals dealing with the assessment and care of the postoperative spine.

## 2. Imaging Methods

Imaging plays a significant role in guiding the physician during the preoperative and operative period, where the imaging exam can correctly evaluate the disease and treatment. Postoperative imaging techniques for the spine include X-rays, CT, and MRI, depending on the type of initial surgery and the patient's symptoms. The postoperative imaging modality depends on the clinical presentation and extent of the disease. Proper sequencing and selection of imaging techniques are essential to evaluate instrument position, fusion status, decompression success, and complication assessment [3, 4].

### 2.1. Radiography

Plain film radiography has almost no use in the diagnostic workup of the noninstrumented postoperative spine [2]. However, plain radiography allows hardware assessment, implant and screw loosening, implant migration, and spinal alignment [5]. Anteroposterior and lateral projections are conventionally performed; if possible, they also include upright weight-bearing position images, and extension and lateral bending may detect instrumentation instability [6]. Radiographs have limited soft tissue evaluation, low resolution, and only bidimensional features [7].

**Figure 1:**
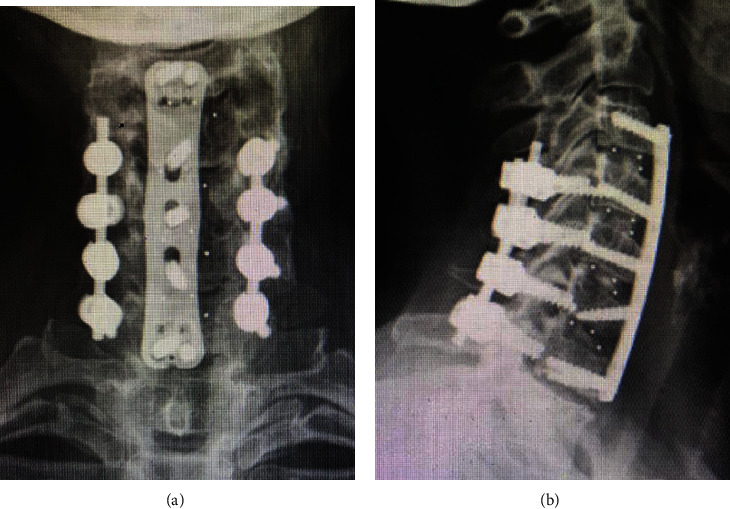
X-rays showing the alignment of the cervical spine using plates and screws. (a) PA view. (b) Lateral view.

**Figure 2:**
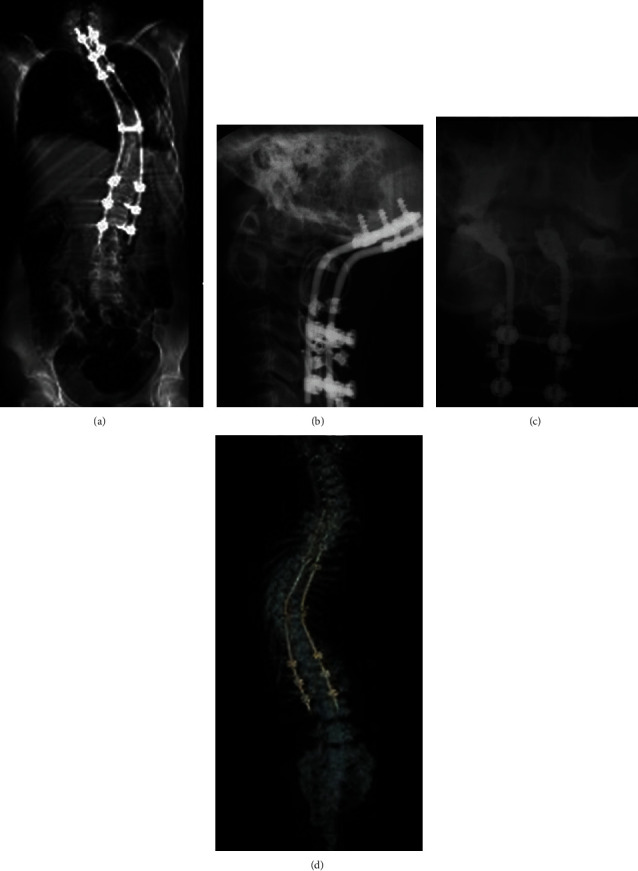
(a–c) Examples of X-rays showing a proper alignment of the spine at the cervical, thoracic, and lumbar levels with stabilization hardware consisting of bilateral rods and transpedicular screws. (d) 3D reconstruction of CT images depicting the hardware located in a case of scoliosis.

Many spine surgeons obtain radiographs at two weeks, six weeks, three months, six months, and one year postsurgery. Reasons to perform postoperative imaging include reassuring the patient about adequate healing and surgery success, identifying asymptomatic hardware migration or failure, and documenting clinical status in the medical record [8]. Figures 1 and 2 present some examples of X-rays used to assess the alignment of hardware after spine surgery.

Others believe routine postoperative radiographical imaging has minimal value for asymptomatic patients [5] and rarely alters the course of treatment. However, it can help determine what additional testing is needed, usually MRI or CT [9].

### 2.2. Ultrasound

**Figure 3:**
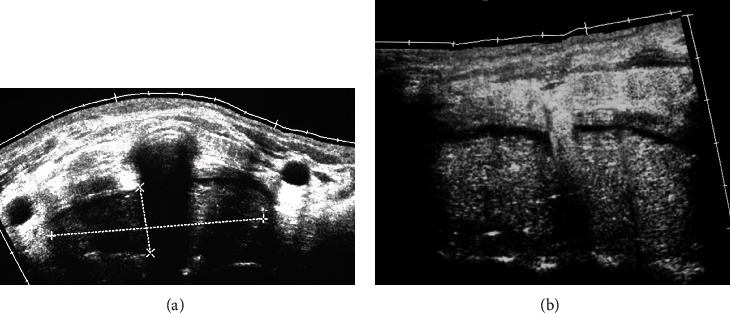
(a, b) Transversal and longitudinal images of ultrasound depicting a serosanguineous fluid collection at the cervical spine in a patient that underwent cervical spine surgery.

Ultrasound (US) can generate images of deep structures without ionizing radiation. Surgeons use this technique to evaluate spinal decompression, lumbar disc degeneration, herniation, and muscle tears in children. It is limited by interoperator variability, artifacts, and elevated false-positive rates when assessing nerve root or spinal facet inflammation [10]. This method is not used commonly for evaluating complications, while abscesses or fluid collections are easily detected [11].  Figure 3 shows a hematoma observed in US after cervical spine surgery.

### 2.3. Computed Tomography

**Figure 4:**
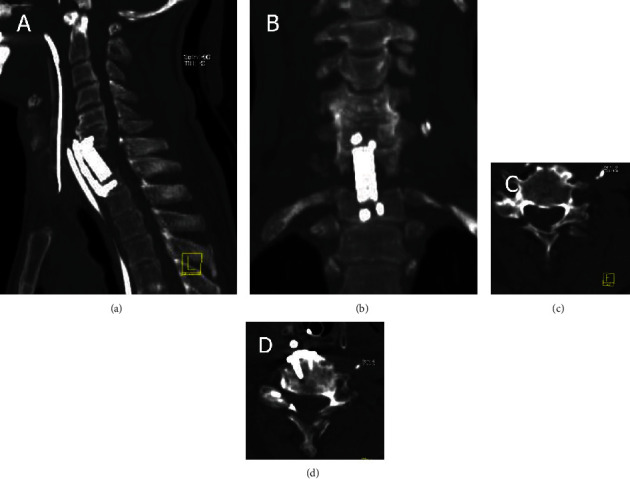
2D multiplanar CT reformation of the cervical spine in the bone window algorithm. (a, b) Sagittal and coronal views depict the location of plates and screws at the C6-T1 level. (c) Axial view above the fixation level. (d). Axial view at the T1 level.

After spine surgery, CT helps assess instrumentation mispositioning, disruption, and loosening; additionally, it can determine bone continuity and graft fusion. Assessment or fusion surgery can identify pseudarthrosis and infection. Helical CT detects spinal and foraminal stenosis. Intravenous contrast enhancement is helpful to differentiate fibrosis from recurrent herniation [12].  Figure 4 displays MIP-like reconstructions with thickened slices of the cervical spine depicting fixation at the spine using plates at the C6-T1 level.

**Figure 5:**
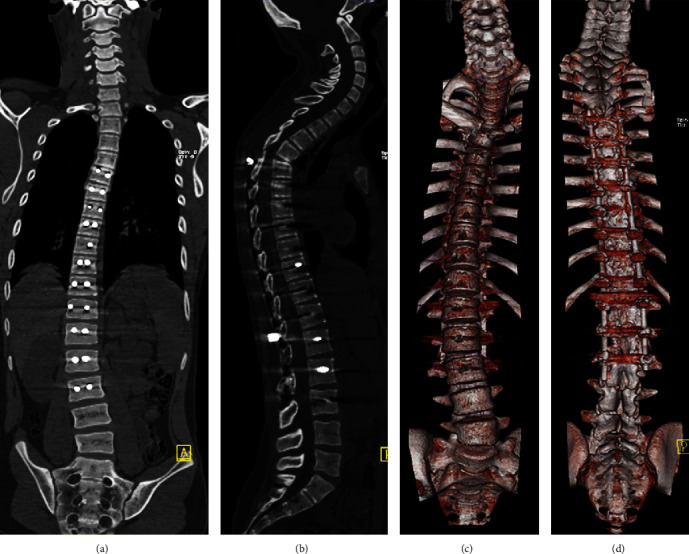
2D and 3D reconstructions of the spine in the bone algorithm show alignment and implant positions after thoracic and lumbar spine surgery. (a, b) Coronal and sagittal views. (c, d) Anterior and posterior aspects of 3D CT reconstructions.

Multidetector CT (MDCT) evaluates spinal alignment and integrity, implant position, fusion progress, and bone graft incorporation in the facet [13].  Figure 5 shows some examples of multiplanar and 3D reconstructions of the entire spinal alignment and implant positions after thoracic and lumbar spine surgery.

**Figure 6:**
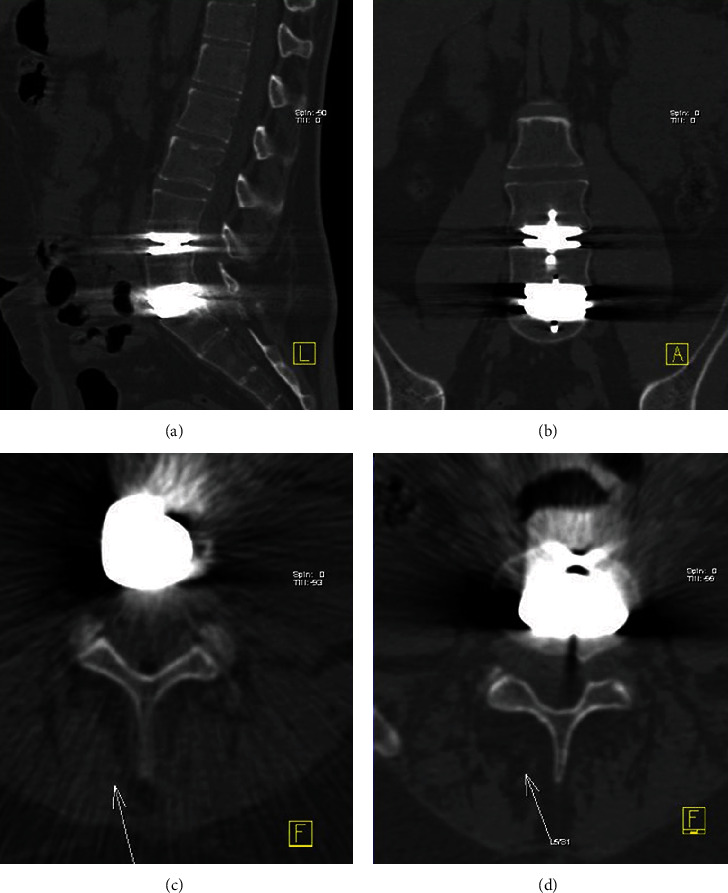
Examples of CT beam hardening artifacts caused by a metallic prosthesis in the lumbosacral (a, b) and cervical (c, d) spine.

MDCT reduces scanning time and motion artifacts; equivalent image resolution is created by using very thin sections, and higher milliamperage allows greater penetration of hardware [14]. Although CT is efficient in evaluating the postoperative spine, it is sometimes limited by the artifact generated by the beam hardening phenomenon when using metallic hardware. Metal attenuation is depicted as dark and bright strips that diminish the visualization of bone, soft tissues, and hardware [15].  Figure 6 shows CT beam hardening examples in the cervical and lumbosacral spine.

Titanium implants have a lower beam attenuation coefficient; hence, an artifact is less common.

**Figure 7:**
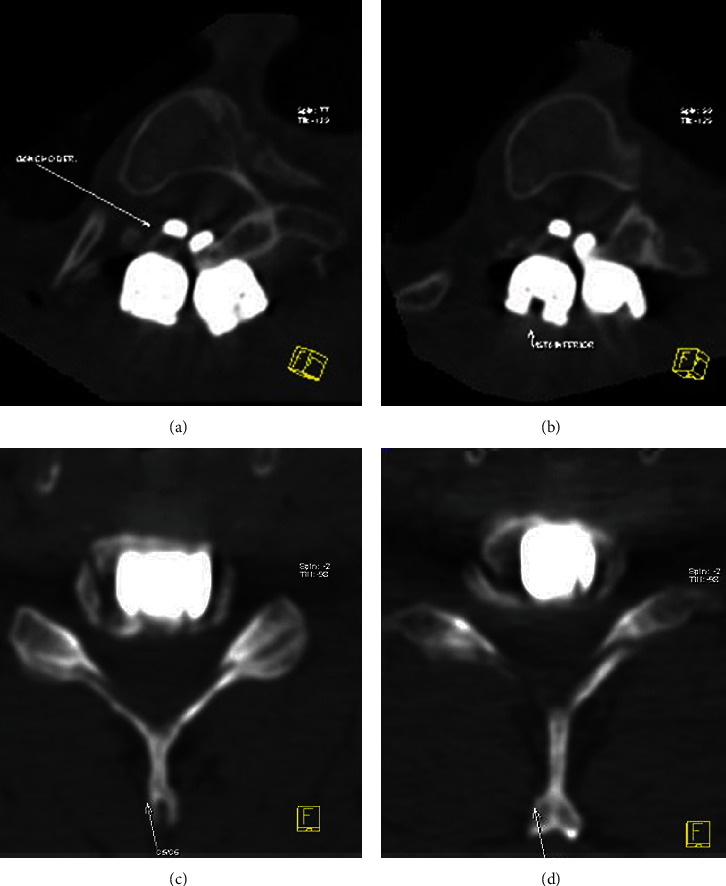
Examples of lower beam attenuation with reduced artifacts by using titanium implants.

The beam must be located perpendicular to hardware to grant the beam to traverse the metallic implant at the smallest diameter [12].  Figure 7 depicts a lower beam attenuation with reduced artifacts achieved by using titanium implants.

Metal artifact reduction techniques are classified into standard (optimized acquisition parameter) and advanced (dedicated acquisition and postprocessing methods). Standard reduction recommends increased kilovolt peak (tube potential), milliampere second (tube current), beamwidth reduction, thin slices, narrow collimation, and extended CT scale. In comparison, advanced techniques advise monoenergetic dual-energy CT and replace corrupted raw data [16].

### 2.4. Magnetic Resonance Imaging

In most cases, MRI is the imaging gold standard for evaluation of the postoperative spine, suspected complications, and recurrent pain after the surgery [17, 18]. MRI facilitates imaging due to the high resolution of soft tissue, which allows the assessment of the nerve roots, spinal cord, and neuroforaminal and spinal canal [19, 20]. Also, it facilitates discrimination between normal postoperative imaging findings and complications such as inflammation, bleeding, and infection [18].

Metal fixation devices distort the local magnetic field, called artifacts. Artifacts originate from magnetic susceptibility caused by the variability of hardware magnetic properties concerning surrounding soft tissues [15]. Ferromagnetic implants like stainless steel produce more artifacts compared to nonferromagnetic implants such as titanium or tantalum. Nonferromagnetic materials originate radiofrequency artifacts but may still obscure regional anatomy [21].

**Figure 8:**
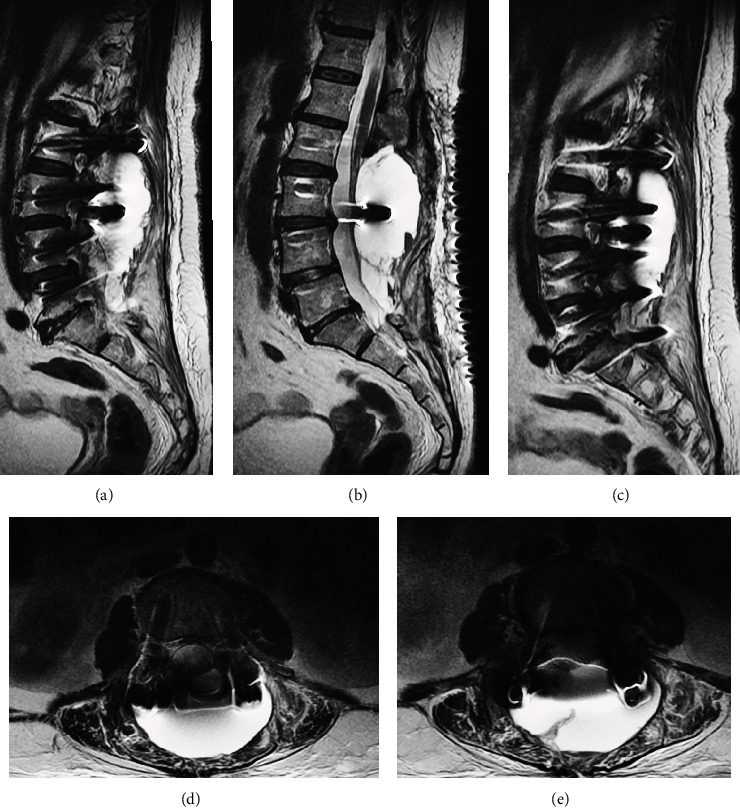
Lumbar spine MRI shows artifacts produced by metal hardware in a patient with a large pseudomeningocele. (a–c) Sagittal view. (d, e) Axial view.

Artifact reduction techniques that can be implemented include encoding phase direction parallel to the hardware long axis in both the axial and sagittal planes, using a fast spin echo sequence (for short echo spacing maintenance), increasing bandwidth, decreasing voxel size, decreasing field strength, using short tau inversion recovery (STIR) techniques for regional fat suppression, and using view angle tilting [22, 23]. Specific sequences for metal artifact reduction are MAVRIC-SL (multiacquisition variable resonance image combination selective) and SEMAC (slice encoding metal artifact correction) [24].  Figure 8 shows some examples of artifacts produced by metal hardware in a lumbar spine MRI pseudomeningocele.

Currently, there is no standardized MRI routine for the spine, but it usually includes the T1 and T2 axial images, sagittal T2 fast turbo spin echo, T1 STIR images, and T1 short echo after gadolinium administration [25]. On T2, normal intervertebral discs are bright (relatively high signal intensity); sagittal T2 with relatively short echo train lengths (<10 ms) is preferable to diagnose disc degeneration. Sagittal and axial T2 also show the spinal cord and the nerve roots of the cauda equina [2]. On T1, fat in the lumbar spine is very bright and contrasts nicely with the dural sac and intervertebral disc; the high signal intensity of normal epidural fat contrasts nicely with postoperative epidural fibrosis, which is dark. Postgadolinium T1 images are mandatory in differentiating scar tissue (fibrosis) from recurrent disc herniation [26].

An additional coronal T2-weighted image could be obtained to depict lumbosacral anatomy and detect disc herniation in the cervical spine. An axial echo sequence is performed to differentiate material from osteophytes [24].

The T1 sequence helps bone assessment; axial images can evaluate altered or absent areas of bone; epidural fat is very bright on T1. The T2 sequence depicts the shape of the thecal sac, spine, nerve roots of the cauda equina, and regions of compression by scar tissue. Moreover, in degenerated intervertebral discs, the relaxation time shortens, and the discs become darker [27, 28].

**Figure 9:**
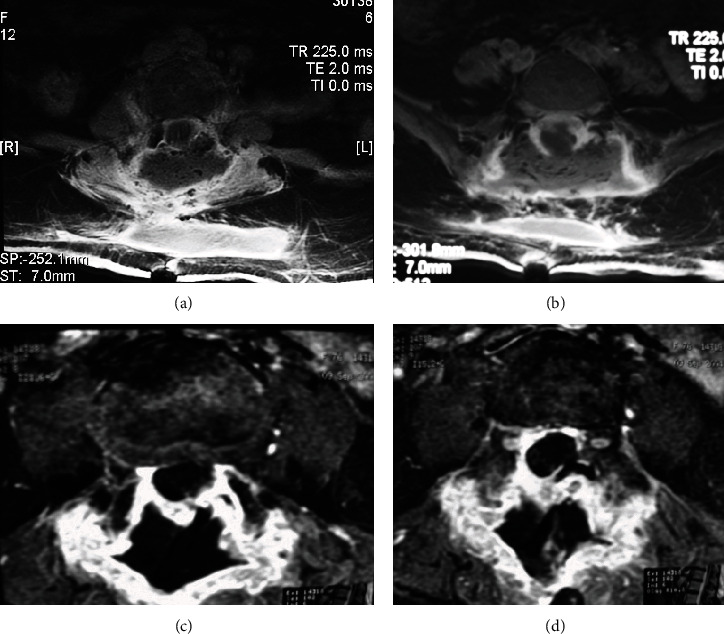
(a–d) Postgadolinium MRI is showing abscesses at the lumbar spine. (a, b) Serosanguineous fluid collections extend to the subcutaneous tissue. (c, d) Intense enhancement of the dural sac.

The postcontrast pattern of nerves, meninges, joints, and soft tissue must be evaluated. The postgadolinium fat-saturated T1 sequence can evaluate discectomy, infection suspicion, or disguise between recurrent disc herniation and scar tissue [29]. At a minimum, in the postoperative period, one fat-suppressed image is needed to evaluate collections [30].  Figure 9 displays abscesses at the lumbar spine in the postgadolinium T1 sequence.

### 2.5. CT vs. MRI

Up to two-thirds of spine imaging assessments may be inappropriate, whether it is CT or MRI; high imaging rates relate to high surgery rates. Factors like lawsuits need visual evidence of surgery success, and financial motivation could explain the underlying cause of inappropriate imaging [31].

In fixation spine surgery, metallic hardware is visualized with CT to evaluate neural compression and fusion status [32]. Moreover, MDCT has high spatial and temporal resolution showing bone, soft tissues, disc bulging, and hypertrophy of the ligamentum flavum [33]. On the other hand, the radiation dose associated with a spinal CT is elevated. It varies according to the spinal section: 5 mSv for cervical (400 radiographs), 7 mSv for lumbar (500 radiographs), and 8 mSv for thoracic (550 radiographs) [34].

As a person gets older, the harm associated with radiation diminishes due to the reduced lifetime cancer risk. At the same time, younger patients have an increased risk of cancer-related imaging radiation. The harm may exceed any benefit [35]. An increasing amount of evidence links CT scans to an elevated risk of neoplasia later in life [34, 36, 37].

In contrast to CT, MRI is not associated with the risk of cancer development; this imaging modality can assess neural elements, soft tissues, vertebrae, and adjacent segments. Disadvantages are related to cost, access, and the potential for heating, displacement, or dysfunction of metallic implants [38].

MRI has been tested for fusion assessment in patients with posterior lumbar interbody fusion (PLIF); it was found that the coronal plane best demonstrates bone fusion [39]. Another study showed that MRI could accurately detect displaced screws. Future research has to investigate the optimal metal artifact reduction sequence for spine fixation on MRI [40].

Routine surveillance scanning should be discouraged when symptoms are not present; patients should be informed about the risks and benefits when a CT is required. Currently, novel materials for instrumentation are being tested [32]; for example, polyether ether ketone (PEEK) is a polymer that can be imaged on magnetic resonance with very reduced metal artifacts around the implant region [41, 42].

### 2.6. Radionuclide

A radionuclide scan is sensitive for bone metabolism alterations, demonstrating pathologic changes before they appear on anatomic imaging, for example, technetium-99m (99mTc) [43]. As a complementary technique to MRI, the use of 99mTc hydroxymethylene diphosphonate combined with gallium-67 helps diagnose postoperative spine infection [44].

### 2.7. Bone SPECT/CT

Single Photon Emission Computed Tomography (SPECT) provides metabolic activity and anatomical localization; it has high sensitivity in identifying sources of pain [45] and postoperative complications like pseudarthrosis, radiculopathy, adjacent segment degeneration, hardware failure, and radiographically occult fractures, although it is not possible to assess disc herniation, root compression, stenosis, and listhesis [43].

### 2.8. Positron Emission Tomography

Positron emission tomography (PET) shows the radioactivity of photons emitted by radiotracers [46]. Fluorine-18 sodium fluoride (18F-NaF) can evaluate the postoperative cervical spine; successful fusion has lower NaF uptake and less pain [47]. The use of 18F-fluorodeoxyglucose (FDG) may identify inflammation, infection, and spondylodiscitis [48] and also depicts high-resolution images with an adequate radiation dose; the drawback includes limited anatomic information, not widely available, and expensive [49].  Table 1 summarizes the main advantages and disadvantages of the imaging modalities used in the postoperative spine.

## 3. Classification of Postoperative Spine Complications

**Figure 10:**
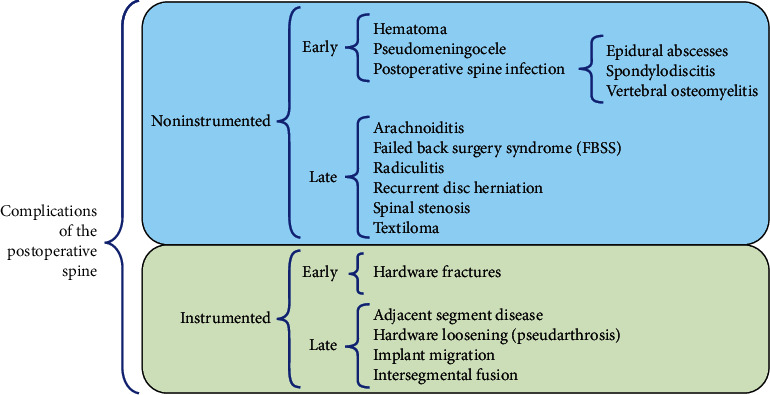
Classification of postoperative spine findings grouped by *noninstrumented* and *instrumented spine complications*.

Imaging is the basis of surveillance for potential complications resulting from surgical procedures. After spine surgery, the patient may have a complete resolution of the low back pain. Otherwise, the patient could experience persistent pain due to complications, which vary depending on the type of instrumentation, the approach, and the patient's underlying condition. When a complication is suspected, it must be discussed with the spine surgeon as soon as possible. Physicians should correlate the clinical presentation of the complications with adequate imaging studies. Complications are classified into *noninstrumented* and *instrumented spine complications*; both groups can be subclassified in early and late complications [1, 2, 4, 50].  Figure 10 presents a classification of postoperative spine findings grouped by *noninstrumented* and *instrumented spine complications*.

### 3.1. The Noninstrumented Spine

#### 3.1.1. Early Complications (6 to 8 Postsurgical Weeks)

**Figure 11:**
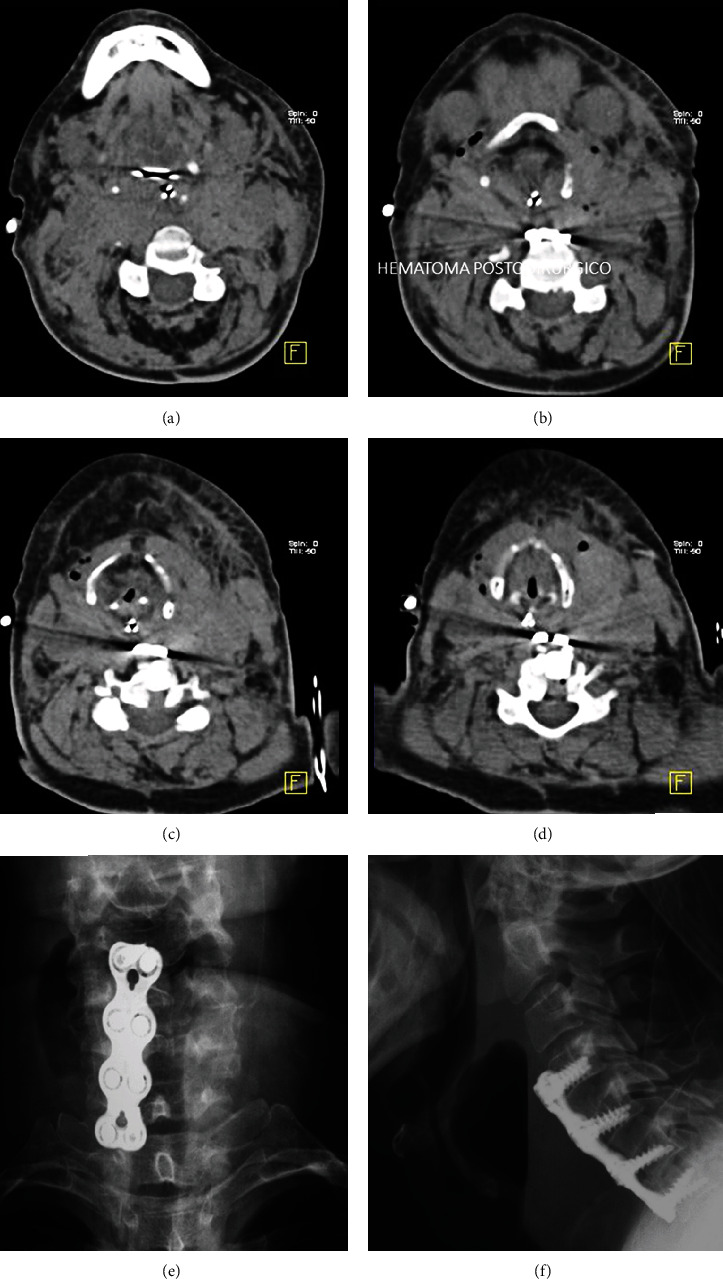
(a–d) CT of the cervical spine. Axial view of hematomas in the anterior aspect of the cervical spine. (e, f) Anterior and lateral views of the cervical spine showing hematomas anterior to a cervical plate.

*(1) Hematomas*. Hematoma is an extravascular collection of blood that extends into the spinal canal or outside. Depending on the time of evaluation, hematomas may present differently on imaging assessment. On CT analysis, during the acute phase, they are isodense to hyperdense [51], in the subacute period isodense, and at the chronic stage hypodense.  Figure 11 shows the CT and X-ray appearance of hematomas in the anterior aspect of the cervical spine.

While on MRI, hematomas are observed on the acute phase as isointense to hypointense on T1 and hypointense to hyperintense on T2; mild contrast enhancement is seen at the periphery; in the subacute period, the intensity of the signal on T1 and T2 is increased with moderate peripheral enhancement; during the chronic stage, both T1- and T2-weighted sequences depict hypointense signal intensity. Collections are best-evaluated on sagittal and axial planes [17, 25]. A large epidural hematoma usually presents variable degrees of neurologic impairment, requiring prompt surgical evaluation, while hematomas with less than one vertebral segment of extension slowly reabsorb [17, 52].

*(2) Pseudomeningocele*. Pseudomeningoceles are an anomalous extradural collection of cerebrospinal fluid (CSF) resulting from the meningeal defect created when CSF extravasates through a dura-arachnoid generally as a result of incidental durotomy during surgery; a cyst is formed inside the wound, in a fibrous capsule. Pseudomeningoceles more than 8 cm in size are described as giant pseudomeningoceles and those more than 5 cm as large pseudomeningoceles [53, 54].

The cerebrospinal fluid signal intensity is matched on T1-, T2-, and diffusion-weighted MRI; sagittal and axial planes on the T2 sequence help to picture its communications.  Figure 8 depicts an example of a large meningocele, as seen in a lumbar MRI.

When contrast is administered, it shows mild peripheral enhancement near the laminectomy site. If contrast enhancement is augmented, clinicians must suspect infection [17].

*(3) Postoperative Spine Infection (Spondylodiscitis, Osteomyelitis, and Epidural Abscesses)*. Infections occurring at the surgical site are the most common cause of morbidity following spinal procedures, ranging from 0.09% to 16% [55], resulting in prolonged hospitalization, wound debridement, hardware failure, revision surgical procedures, implant removal, and long-term use of intravenous antibiotics [56].

**Figure 12:**
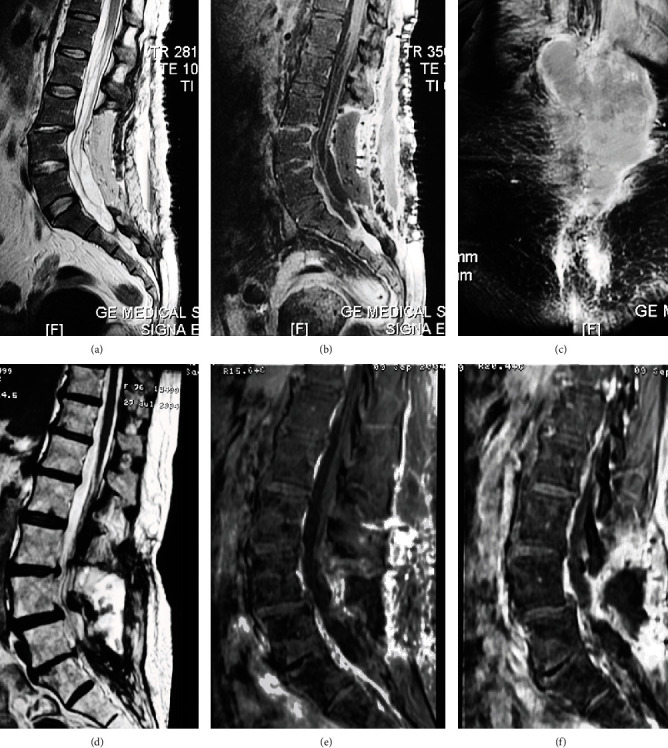
(a, d) Sagittal view of the T2 sequence. (b, e, f) Postgadolinium T1 sequence with fat saturation. (c) Coronal view. Postgadolinium T1 with fat saturation depicting spinal abscesses with extension to a subcutaneous collection. Lumbar infection examples of paraspinal abscesses associated with osteomyelitis and epidural abscesses.

Infections are classified as superficial or deep. Superficial infections are confined to the skin or subcutaneous tissues without fascia involvement, manifesting with pain, inflammation, and increased local temperature. While deep infections affect the fascia or muscle, for example, discitis, osteomyelitis, and epidural abscesses [57], these are related to higher treatment costs and loss of productivity [58].  Figure 12 displays lumbar infection examples of paraspinal abscesses associated with osteomyelitis and epidural abscesses.

The most common pathogens that cause infections are *Staphylococcus aureus*, *Staphylococcus epidermidis*, and *Enterococcus faecalis* [59]. The risk of infection is increased by a posterior surgical approach, instrumentation, bone allograft, blood transfusion, and longer surgical time [60]. The diagnosis is formulated on clinical presentation, laboratory parameters, and radiological findings; persistent elevation for more than two weeks of C-reactive protein is the most sensitive indicator [61].

Plain radiography usually takes about two to four weeks to detect findings following symptom manifestation; disc collapse is an early infection sign. As the infection progresses, a slight decrease in bone density is seen [62].

CT can be done at an early stage in patients with hardware. However, image quality may be affected in the presence of instrumentation; infection is detected as lucency around orthopedic implants [57]. It also shows areas of bone destruction, erosive changes at the endplates, disc space narrowing, and soft tissue collections [62].

**Figure 13:**
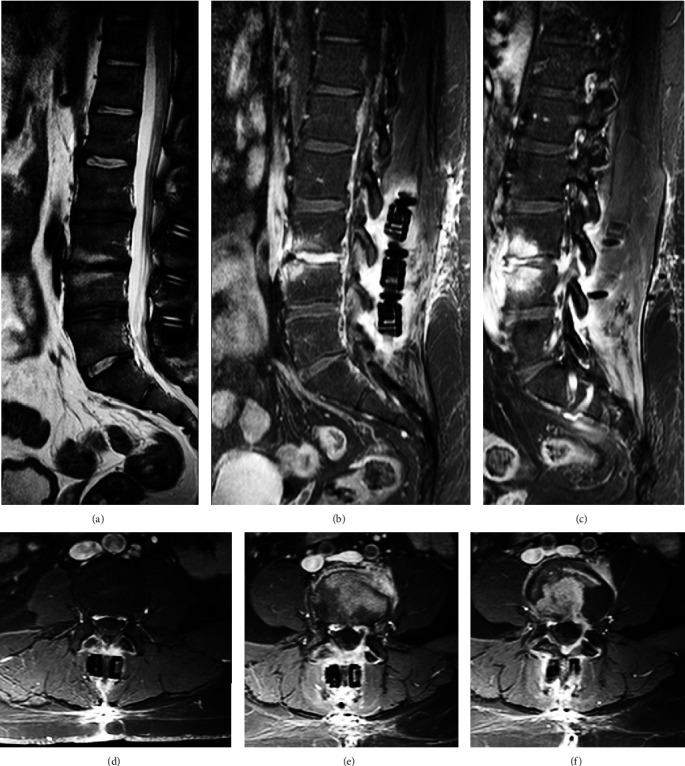
Examples of septic spondylodiscitis on MRI. (a–c) Sagittal view of the lumbar spine. There is a hyperintense signal of the subchondral endplates of L3-L4 on T2 and enhancement after gadolinium administration on T1. There are three consecutive interspinal cages in the interspinal space of L3-L5 surrounded by intense soft tissue enhancement. (d–f) Axial view at the intervertebral level of L3-L5 showing different aspects of discitis and paraspinal tissue enhancement (white arrows).

MRI is the preferred tool to visualize infection lesions of the spine. It depicts subchondral endplate erosions on T1 images; infiltration of the bone marrow is hypointense on T1 and hyperintense on T2. Edema and purulent material are seen as hypointense on T1 and hyperintense on T2, and an increase in postcontrast signal intensity properly detects discitis [63]. Septic spondylodiscitis must be suspected when a contrast-enhanced soft tissue mass is located at the paravertebral or epidural space, in which case a spine biopsy is required [19].  Figure 13 depicts examples of septic spondylodiscitis on MRI.

Spondylodiscitis occurs in about 0.4% of patients in the cervical spine [64] and up to 3% in the lumbar spine [65]. Key MRI findings for diagnosis include [66] absence of peridiscal marrow changes (low signal intensity on T1-WI and high signal intensity on T2-WI) and absence of enhancement of the intervertebral disc space; on the other hand, an enhanced soft tissue mass surrounding the affected spinal level in the perivertebral and epidural spaces is highly suggestive of septic spondylodiscitis.

Radionuclide imaging with gallium-67 has shown earlier detection than CT and plain radiography; it shows focal uptake-increased areas suggestive of infection [67].

#### 3.1.2. Late Complications

**Figure 14:**
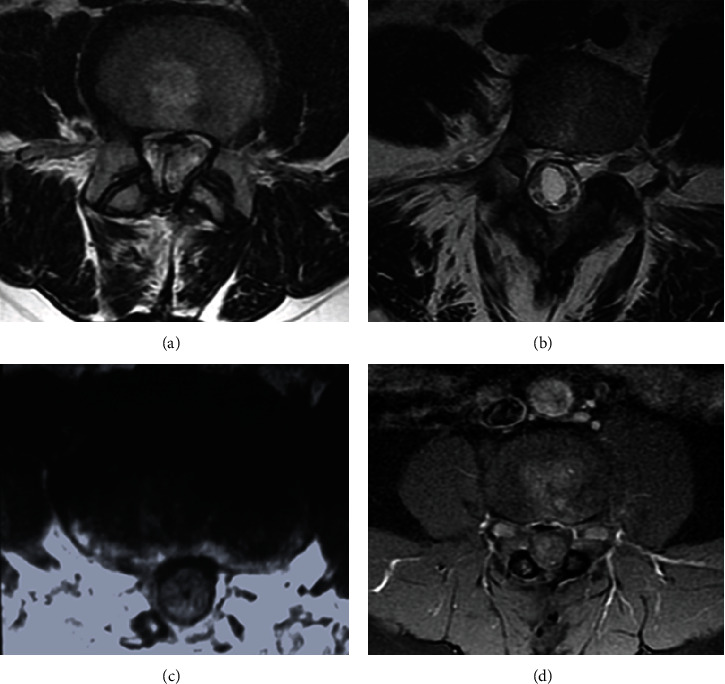
Axial view of lumbar MRI showing different patterns of arachnoiditis. (a, b) T2 sequence showing clumped-up roots in the thecal sac. (c, d) T1 shows an axial view of the lumbar spine in the final stage of the inflammatory response and a swelling mass that occupies the dural sac.

*(1) Arachnoiditis*. The initial inflammation of the arachnoid with swelling of the nerve root, followed by collagen deposition and nerve root adherence, is known as arachnoiditis [68]. On CT myelography, two slightly different patterns could be identified; the first type is depicted as an “empty thecal sac.” In contrast, the second type has diluted filling defects inside the thecal sac [69]. On MRI, the most efficient method to assess arachnoiditis is the acquisition of the unenhanced axial T2 fast spin echo; three patterns are identified: clumped or knotted nerve roots, adhesions of the nerve roots to the walls of the thecal sac that originates an empty sac, and an intrathecal mass with a broad dural base that represents a large group of matted roots [70]. Postgadolinium enhancement of the intrathecal roots and meningeal scarring may or may not be observed [71].  Figure 14 depicts lumbar MRI in different patients with arachnoiditis of the lumbar spine.

*(2) Failed Back Surgery Syndrome (FBSS)*. It is defined as pain of unknown origin that persists or appears after spine surgery is performed to treat pain in the same area [72, 73]. Some incidence studies reported values between 10% and 40% in lumbar laminectomy, 8.84% after lumbar microdiscectomy, 25% to 35% of cases for decompressive surgery, and up to 19% in the two-year follow-up [74].

The patient complains of intractable pain and various degrees of functional incapacity following spine surgery. Identifiable causes include recurrent or residual disc herniation, arachnoiditis, radiculitis, spinal or spinal neural foraminal stenosis, and especially failure to correctly identify the structural source(s) of pain [75].

*(3) Radiculitis*. This complication is identified by pathological gadolinium enhancement after six months postsurgery of the intrathecal nerve roots, resulting from the disruption of the blood-nerve barrier during the surgery or persistent trauma. If a radicular infection is suspected, MRI postcontrast fat-saturated T1 sequences depict augmented root contrast enhancement [20, 76].

*(4) Recurrent Disc Herniation*. It may be made up of disc material, cartilage, or bone. It is more frequent in the lumbosacral region [26]. In residual disc herniation, there is no central enhancement; on the other hand, in epidural fibrosis, there is a uniform enhancement of scar tissue in the anterior, lateral, or posterior epidural space [77].

**Figure 15:**
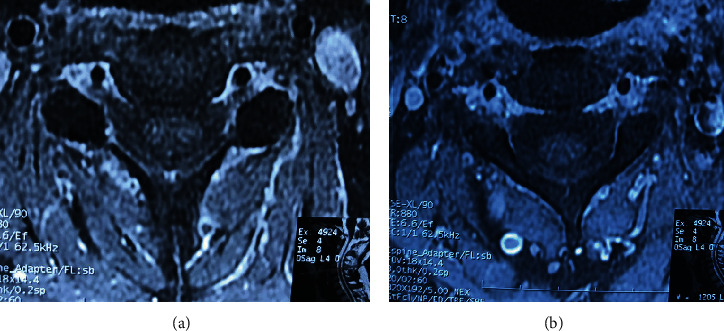
T1, axial MRI showing spinal canal stenosis.

*(5) Spinal Stenosis*. Spinal stenosis originates from the augmented degeneration of intervertebral discs (loss of disc height, annular lesion, and osteophyte formation) after spine surgery. MRI is the ideal diagnostic tool [78]. It is visualized as the sclerotic bone distinguished by low signal intensity on T1 and T2 images. Foraminal stenosis is easily shown in the parasagittal view [12].  Figure 15 shows an axial MRI image that evidences spinal stenosis.

*(6) Textiloma*. It is a surgical sponge or “cottonoid,” accidentally left behind in a surgical wound. Textilomas contain a barium sulfate-marking filament, visible on radiographic examinations but not visible on MRI. Cases of textilomas associated with spine surgery are few in comparison with abdominal or thoracic interventions. The treatment of textilomas is the surgical elimination of the foreign body [79].

### 3.2. The Instrumented Spine

#### 3.2.1. Early Complications

*(1) Hardware Fractures*. Continuous stress and fatigue are related to enhanced spinal motility. If the tip of the edges of the instrumentation hardware is prominent, this can lead to chronic inflammation, pain, and even necrosis of the adjacent tissue; in this scenario, the removal of synthesis material is indicated [80]. Screw fractures can be detected on conventional radiographs, but the detection of fractures can be challenging when there is no displacement of the fractured components; CT offers the best visualization of hardware despite artifacts produced because each instrument can be scrutinized in multiple planes and without overlap [81].

#### 3.2.2. Late Complications

*(1) Adjacent Segment Disease*. This entity is due to accelerated degeneration of discs adjacent to a fused level, the result of soft tissue disruption close to surgery, and increased intradiscal stress on both the upper and lower adjacent levels; the pressure intensifies during flexion and extension movements. The spinal angulation must be preserved after surgery; otherwise, malalignment contributes to the motion segment. MRI is most sensitive in assessing degenerative disc changes (signal change, disc height reduction, herniation, and longitudinal ligament calcification), and CT is more accurate when evaluating osteophytes and longitudinal ligament calcification. It should be noted that surgeons will not decide to operate because of osteophytes, but tissue compression will be the deciding factor [82].

**Figure 16:**
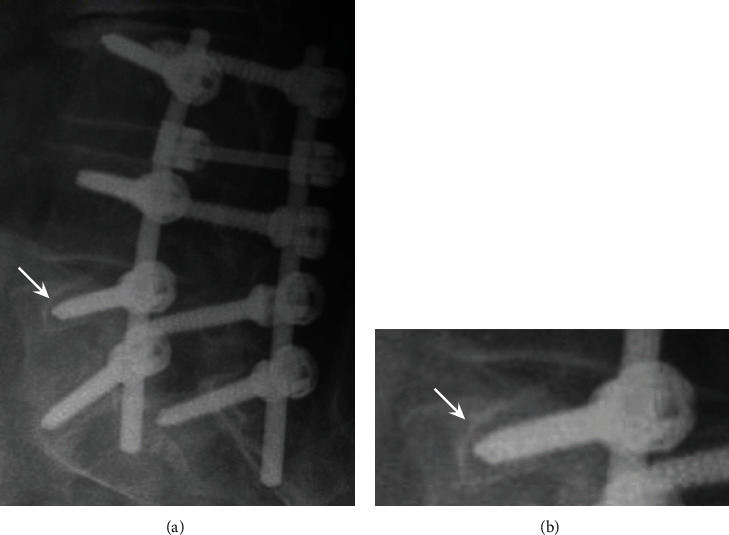
(a, b) Radiography of the lumbar spine with resorption of bone in the vertebral body of L5 leading to hardware loosening.

*(2) Hardware Loosening (Pseudarthrosis)*. Resorption of bone that surrounds screws and implants leads to hardware loosening. Here starts a vicious cycle, in which loosening increases movement, which promotes further bone resorption and finally screw pullout or vertebral fractures. On conventional radiography and CT, the loose material is identified as a lucent halo or rim around the perimeter of screws or plates > 2 mm; every evaluation must be compared with previous imaging [6, 80, 81].  Figure 16 shows radiography of the lumbar spine with resorption of bone in the vertebral body of L5 leading to hardware loosening.

**Figure 17:**
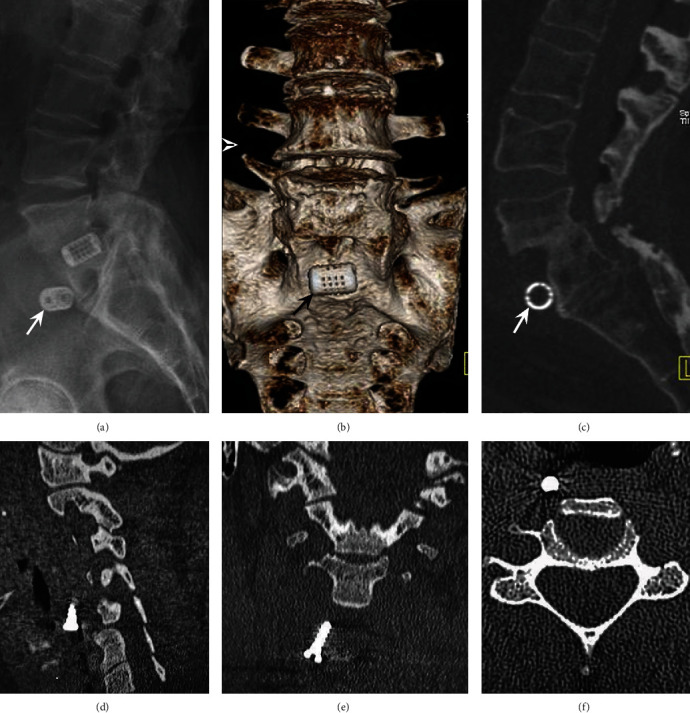
Examples of migration of spinal hardware at the lumbar and cervical spine. (a) X-ray, lateral view of the lumbar spine. (b) 3D CT reconstruction of the sacrum frontal view. (c) Multiplanar CT reconstruction, sagittal view using the bone window. (d) Multiplanar CT, sagittal view showing anterior displacement of a cervical screw at the level of C5-C6. (e) Multiplanar CT, anterior oblique view shows the right displacement of a screw outside the cervical spine. (f) Multiplanar CT, axial view showing anterior and lateral displacement of a cervical screw.

*(3) Implant Migration*. When biomechanics of the spine are locally modified due to hardware loosening, infection, or malignancy, screws and rods and cages can migrate from their position, resulting in damage to adjacent tissue or piercing essential structures [81]. To evidence implant migration, plain radiographs and CT are the best imaging choice [83]. Risk factors include multilevel fusion surgery, especially at the L5-S1 level, instability, scoliosis, osteopenic vertebrae, wrong size of hardware, positioning, and type of material [84].  Figure 17 evidences examples of migration of spinal hardware at the lumbar and cervical spine.

*(4) Intersegmental Fusion*. It is visualized as the visual obliteration of the cortical vertebral endplates and loss of the *graft*-*host* interface between the bone implant and the native vertebral bone. At least 6 to 9 months from the time of surgery are necessary to develop solid intersegmental fusion to be seen radiographically. On MRI, solid fusion sometimes leads to conversion of previous Modic type I changes to fatty Modic type II changes confirming immobilization of the fused segment [2].

## 4. Follow-Up of the Postoperative Spine

After the surgery is done, immediate postoperative imaging is performed to assess the placement of hardware, decompression, alignment of the spine, and complications; generally, any additional imaging is misleading and unnecessary. Overall, physicians should know about the type and time point of surgery, instrumentation specifications, biomechanics, and complications related to the procedure [45, 85].

In case new symptoms manifest, such as intensifying pain, acute neurologic deficit, or signs of infection, additional imaging is required. It is fundamental to compare preoperative and postoperative imaging to assess complications; the imaging should be interpreted parallel with the patient's clinical state [85, 86].

Readers should be aware that for some hospitals, X-ray is still used as the first-line tool in the assessment of the postoperative spine; when complications are suspected, CT is useful when a diagnosis cannot be made on X-ray. However, a CT scan is prone to metal artifacts. Given this spectrum of possible acute and late complications, MR imaging is considered the imaging modality of choice [45, 86].

Imaging of the postoperative spine should be performed depending on the clinical findings:

- Within days of surgery for surveillance, if there is severe pain or acute neurologic deficit
- During the first two months postsurgery for surveillance, when there is a poor postoperative recovery or continuous pain
- During the first year for surveillance, if there is constant pain, and if after years free of complications, pain returns [85]

**Figure 18:**
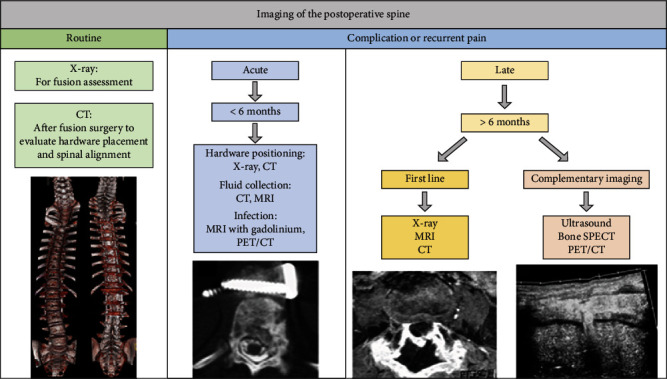
Suggested postoperative spine imaging methods in routine follow-up, complications, and recurrent pain.

A postoperative spine imaging method algorithm in routine follow-up, complications, and recurrent pain is proposed in Figure 18.

## 5. Conclusions

Imaging of the postoperative lumbar spine is a standard procedure in the day-to-day practice of radiologists. They must know the patient's clinical situation and the various surgical techniques and instrumentation material, must be aware of the routine postoperative findings, and must distinguish them from pathologies encountered following surgery. The imaging report in the postoperative spine should contain four elements [25]: confirmation of the level(s) treated in comparison with preoperative examinations; interpretation of normal postoperative changes; detection of early and late complications, according to the clinical findings; and evaluation of surgical hardware (interbody implants, pedicle screws).

Regardless of the full range of inventions in imaging technology, postoperative spine imaging remains a difficult task. A systematic approach is critical to offer an accurate diagnosis for the outcome and management of the patient. It is essential to compare the advantages and disadvantages of the imaging modalities to choose those that provide more accurate spinal status information during the follow-up.

## Figures and Tables

**Table 1:** Advantages and disadvantages of postoperative imaging modalities.

Imaging modality	Advantages	Disadvantages
Ultrasound	No ionizing radiation Fluid collection Identification [10, 11]	Intraoperator variability Inferior 3D imaging Limited use in the assessment of postoperative complications [10, 43]

Radiography	Hardware assessment: alignment, loosening, and migration [5] Dynamic image: flexion, extension, and lateral [6]	Nondiagnostic in some scenarios Metal artifact Low three-dimensional features and soft tissue resolution [7]

Computed tomography	Excellent bone detail Superior 3D imaging Assessment of instrumentation [12] Fusion progress and bone graft incorporation [13]	Metal artifact caused by a prosthesis [15] Cannot differentiate acute from chronic changes Overestimation of lucencies High radiation dose [34]

Magnetic resonance imaging	Superior for evaluating discs, soft tissues, and intradural and cord pathologies Detection and monitoring of infection or fluid collections No ionizing radiation [18–20]	Magnetic artifacts [15, 21] Known contraindications: pacemaker Unable to assess the cortical bone [43, 45]

SPECT/CT	High sensitivity Osteoblast activity assessment Reduced metal artifacts [43, 45]	Low specificity Unable to assess disc herniation, root compression, stenosis, or listhesis [43]

Positron emission tomography	Detection of inflammation, infection, and spondylodiscitis [48] High-resolution images Adequate radiation dose [49]	Limited anatomic information Not widely available Expensive [49]

## Data Availability

The data used to support the finding of this study are available from the corresponding author upon request.
